# Research advances in m^6^A methylation and sepsis

**DOI:** 10.3389/fcell.2025.1682283

**Published:** 2025-11-18

**Authors:** Lifan Zhang, Wenjuan Chen, Yafeng Liu, Shujun Zhang, Bingyou Yin, Kaijie Liu, Xinyu Gu, Xinjun Hu

**Affiliations:** 1 Department of Infectious Diseases, The First Affiliated Hospital, College of Clinical Medicine, Henan University of Science and Technology, Luoyang, Henan, China; 2 Henan Medical Key Laboratory of Gastrointestinal Microecology and Hepatology, Luoyang, China; 3 Henan Key Laboratory of Cancer Epigenetics, Cancer Institute, The First Affiliated Hospital, College of Clinical Medicine, Medical College of Henan University of Science and Technology, Luoyang, China

**Keywords:** m6A methylation, sepsis, organ injury, inflammatory response, RNA methylation

## Abstract

Sepsis is an infection-induced syndrome driven primarily by dysregulated host inflammatory responses. This process induces complex physiological changes that provoke systemic inflammation and multi-organ dysfunction, severely threatening survival in advanced cases. N6-methyladenosine (m^6^A), the most prevalent eukaryotic RNA modification, orchestrates crucial regulatory functions across biological processes and is a focal point in epigenetics. This modification is dynamically controlled by three protein classes: writers that catalyze m^6^A deposition, erasers that mediate its removal, and readers that decode modification signals. Substantial evidence implicates m^6^A dysregulation in sepsis-induced multi-organ damage, encompassing cardiovascular dysfunction, acute lung injury, and acute kidney injury. This review synthesizes current mechanistic insights into m^6^A’s role in sepsis pathogenesis. By delineating how m^6^A governs inflammatory cascades and organ injury pathways, we evaluate its therapeutic targeting potential, providing translational frameworks for future research.

## 1 Introduction

Sepsis is currently defined as a life-threatening organ dysfunction caused by a dysregulated host response to infection (Singer et al., 2016). It poses a critical threat to patients due to its high potential to progress to multiple-organ dysfunction syndrome (MODS) and other lethal complications (Wheeler and Bernard, 1999). Globally, sepsis accounts for 20% of annual deaths. (Rudd et al., 2020).Its high mortality rate correlates strongly with heterogeneous manifestations, primarily involving damage to the heart, lungs, kidneys, and other organs. (Wheeler and Bernard, 1999; Borges and Bento, 2024; Gustot et al., 2009; Ricci et al., 2011). The pathogenesis of sepsis is now understood as a dysregulated host response to infection. This response is characterized by a complex and often concurrent interplay between an initial hyperinflammatory phase (frequently manifesting as a “cytokine storm”) (Nedeva, 2021) and a subsequent protracted immunosuppressive state. A critical component of this immunosuppression is the development of immune tolerance, a state of lymphocyte hyporesponsiveness and innate immune paralysis. Key mechanisms include extensive apoptosis of immune cells, T-cell exhaustion, and reprogramming of monocytes/macrophages with diminished antigen-presentation capacity and cytokine production. (Hotchkiss et al., 2013a; Arora et al., 2023). It is precisely this bimodal and dysregulated immune response—the uncontrolled inflammation coupled with compensatory immunosuppression and tolerance—that distinguishes sepsis from an uncomplicated infection, however serious, and underlies the heightened vulnerability to secondary nosocomial infections and later mortality. Consequently, elucidating the pathogenesis of sepsis and identifying targets for early diagnosis and therapeutic intervention have significant clinical implications for improving patient prognoses.

A key clinical indicator of sepsis severity and tissue hypoperfusion is hyperlactatemia, which is particularly prominent in septic shock and strongly correlates with poor outcomes (Garcia-Alvarez et al., 2014). Beyond its role as a metabolic byproduct, lactate is increasingly recognized as a signaling molecule that can influence gene expression through novel epigenetic modifications, such as histone lactylation (Xiong et al., 2022). Gene expression is regulated through heritable, non-DNA-sequence-changing mechanisms across multiple levels. Epigenetic modifications, such as DNA methylation, histone modification, chromatin remodeling, and non-coding RNA (ncRNA)-based regulation, modulate gene activity by altering chromatin accessibility and function (Dai et al., 2024; Bollati and Baccarelli, 2010). In parallel, epitranscriptomic modifications, which refer to post-transcriptional chemical alterations to RNA, represent another critical regulatory layer. Notably, among the >170 identified RNA modifications, m^6^A methylation regulates all phases of the RNA lifecycle (Zaccara et al., 2019)—such as processing, degradation, nuclear export, and translation—thereby modulating RNA expression and function. This modification is dynamically controlled by three protein classes: “writer” (methyltransferases), “eraser” (demethylases), and “reader” (reader proteins) (An and Duan, 2022).

m^6^A methylation has been implicated in diverse pathologies, including acute promyelocytic leukemia, (Wu et al., 2025), ischemic brain injury (Xu et al., 2020), and clear-cell renal carcinoma (Strick et al., 2020; Alhammadi et al., 2025). Recent studies suggest its involvement in sepsis pathogenesis. Analysis of gene-expression datasets from 479 sepsis patients by Zhang et al. revealed three sepsis subtypes characterized by heterogeneity in m^6^A methylation-regulated genes, indicating a link between m^6^A dysregulation and sepsis heterogeneity (Zhang S. et al., 2020). The link between lactate and m^6^A adds another layer of complexity. For instance, Xiong et al. demonstrated that in tumor-infiltrating myeloid cells, lactate induces METTL3 expression via H3K18 lactylation (Galle et al., 2022), and this METTL3-mediated m^6^A modification promotes immunosuppression via JAK/STAT signaling (Xiong et al., 2022) connection suggests a potential mechanism whereby lactate-driven METTL3 induction and subsequent m^6^A deposition may contribute to the dysregulated immune response and immunosuppression observed in septic patients.

This review synthesizes recent advances in m^6^A modification within the context of sepsis, outlining its fundamental biology, examining its mechanistic roles in sepsis-induced MODS, and evaluating its potential as a therapeutic target—ultimately aiming to open novel diagnostic or therapeutic avenues for improving sepsis outcomes.

## 2 m^6^A methylation: molecular mechanisms and functions

Among over 100 identified RNA chemical modifications, m^6^A represents the most prevalent and abundant modification in eukaryotic mRNA. This modification occurs at the N6 position of adenosine residues. (Xu Z. et al., 2025; Cappannini et al., 2024). Research confirms its conservation across diverse species—including plants, humans, *Drosophila*, and other mammals. (Oerum et al., 2021).

Critically, m^6^A modification levels undergo rapid, reversible reprogramming in response to environmental stimuli (Furci et al., 2024; Dierks et al., 2025; Zhang et al., 2025), developmental stages (Li et al., 2022), and RNA metabolic states (Furci et al., 2024; Dierks et al., 2025). This dynamic regulation enables m^6^A to participate extensively in RNA-related cellular processes—particularly differentiation and reprogramming—thereby highlighting its broad relevance to disease pathogenesis (Jiang et al., 2021a).

m^6^A modification is reversible and participates in eukaryotic cell differentiation, proliferation, and apoptosis (Zhang H. et al., 2020). Its regulatory factors fall into three categories: writers, erasers, and readers (as summarized in Table 1).

**TABLE 1:** m6A methyltransferase and organ damage in sepsis.

Type	Factor	Function	Organ damage in sepsis	Reference
m6A Writer	METTL3	Catalyzes m6A modification	SCM	Shen et al. (2023), Wang et al. (2023), Wang et al. (2024), Tang et al. (2024), Shen et al. (2022)
			ARDS/ALI	(Chen et al., 2022)
			SAE	(Wang et al., 2022b)
	METTL14	Form heterodimer with METTL3 to catalyze m6A Modification	SCM	(Wang et al., 2023)
			ARDS/ALI	(Lai et al., 2025)
			AKI	Adedoyin et al. (2018), Yang et al. (2024)
	WTAP	Combine the METTL3-METTL14 catalytic subunits and anchoring them at the nuclear speckle	AKI	(Huang et al., 2024)
	KIAA1429	m6A writer, Recruits and mediates the binding of methyltransferase and specific RNA site	—	—
	METTL16	Modify mRNA and non-coding RNA	—	—
	RBM15B	Binds uridine-rich regions to enable selective methylation	—	—
M6A Erasers	FTO	Stepwise oxidative demethylation; regulates mRNA stability	AKI	Yang et al. (2024), Yang et al. (2021b), Tan et al. (2020)
	ALKBH5	Direct demethylation; modulates mRNA nuclear export	SAE	(Ye et al., 2025)
	ALKBH1	Demethylates noncoding RNAs	—	—
m6A readers	YTHDF1	Promotes mRNA translation	SCM	(Zhang et al., 2022a)
	YTHDF2	Promotes mRNA degradation	—	—
	YTHDF3	Interacts with YTHDF1 to promote mRNA translation or interacts with YTHDF2 to promote mRNA degradation	—	—
	YTHDC1	Regulates pre-mRNA splicing and nuclear export	—	—
	YTHDC2	Improves the translation efficiency oftarget mRNA	—	—
	eIF3	Promotes mRNA translation	—	—
	IGF2BP1/2/3	Promotes the stability and translation of mRNA	ARDS/ALI	(Cao et al., 2024)
			SAE	(Ding et al., 2022)
			Sepsis liver injury	(Sun et al., 2024)
	HNRNPA2B1	Promotes primary miRNA processing and mRNA splicing	—	—

The writers recognize and bind to m^6^A-modified RNA, regulating mRNA stability, translation efficiency, splicing, and nuclear export. This group primarily includes the methyltransferase-like proteins methyltransferase-like 3 (METTL3),METTL14 and Wilms’ tumor 1-associating protein (WTAP). Within this complex, METTL3 serves as the catalytic subunit, while METTL14 provides structural support at the active site (Wang et al., 2016). The readers decode the m^6^A marks and regulate mRNA metabolism through distinct mechanisms. Key examples include YTH domain-containing family proteins (YTHDF1-3 and YTHDC1-2) and eukaryotic translation initiation factor 3 subunit A (eIF3), which recognize m^6^A sites to modulate target RNA function. The erasers remove m^6^A modifications from RNA (32), dynamically controlling modification levels and participating in cell development and stress responses. Major erasers include fat mass and obesity-associated protein (FTO) and alkB homolog 5 (ALKBH5), which mediate m^6^A demethylation (Kapadia et al., 2025).

**FIGURE 1:**
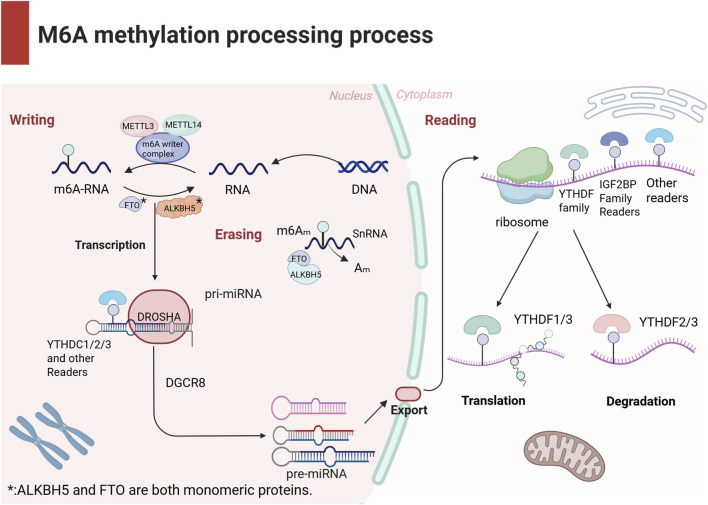
The schematic diagram presents the biological process of m6A modification. m^6^A methylation is a dynamic and reversible modification regulated by three types of factors: Writers, Readers, and Erasers. Writers are responsible for adding methyl groups; Readers recognize the chemical modification and regulate mRNA metabolism through diverse mechanisms; Erasers remove m^6^A modifications from RNA, dynamically modulating m^6^A levels to participate in cellular development and stress responses. These components collectively maintain cellular m^6^A homeostasis and ensure precision in RNA functional regulation.

These regulatory factors cooperate to determine m^6^A homeostasis within cells and ensure the precision of m^6^A methylation, thereby influencing RNA functionality and biological behavior (as illustrated in Figure 1). Research indicates that m^6^A methylation exhibits dynamic regulatory properties, meaning that its regulatory mechanisms may differ across cell types (Ivanova et al., 2017) and physiological states (Li et al., 2022), thus offering new scientific perspectives (Yang B. et al., 2021).

m^6^A methylation critically regulates diverse biological processes (Jiang et al., 2021a). First, it influences gene expression by modulating RNA stability and translation efficiency. For example, m^6^A methylation can dynamically regulate mRNA stability—either promoting degradation or enhancing stability—in a context-dependent manner (Wei, 2024; Bi et al., 2023). Additionally, m^6^A governs the RNA lifecycle through its impact on RNA splicing and nuclear export. In immune responses, m^6^A modifications regulate the effector functions of immune cells, ultimately shaping systemic immunity. Critically, dysregulated m^6^A methylation is mechanistically linked to multiple pathologies, including cancer, cardiovascular disease, and neurodegenerative disease (An and Duan, 2022).

## 3 m^6^A methylation regulates sepsis progression through immune-inflammatory networks

**FIGURE 2:**
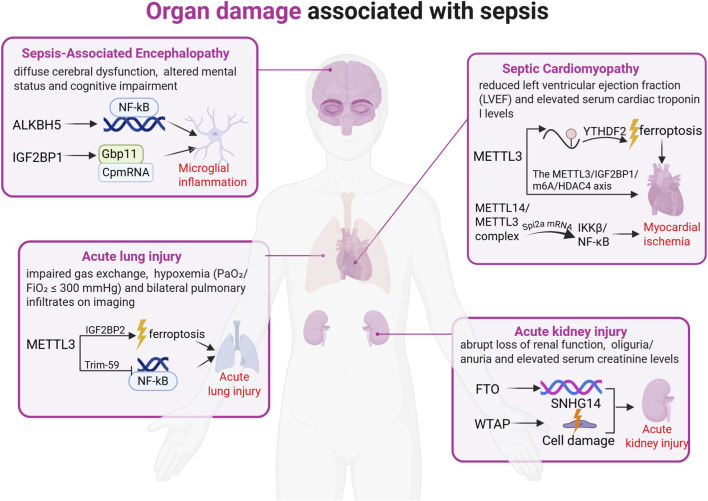
Organ damage associated with sepsis. Severe sepsis is frequently accompanied by organ damage, primarily involving injury to the heart, lungs, kidneys, and brain. Research indicates that sepsis-induced organ damage is driven by aberrant RNA modifications and their regulatory factors, manifested as endothelial cell injury and ferroptosis. The figure above illustrates the role of m^6^A modification in organ damage during sepsis.

Sepsis is a multisystem disorder characterized by high mortality and complex multidimensional clinical and biological features (Singer et al., 2016). Its heterogeneity stems from diverse factors including host genetics, infection etiology, dysregulated host responses, and multi-organ dysfunction (W et al., 2023). Emerging evidence indicates that m^6^A methylation plays a critical role in sepsis pathogenesis. (Zhu et al., 2024). Ge et al. demonstrated that elevated WTAP protein and m^6^A levels correlate strongly with hyperinflammatory responses. Under inflammatory stress, WTAP is upregulated under the regulation of nuclear factor kappa-B(NF-κB) and accelerates the inflammatory response by promoting the expression of numerous pro-inflammatory cytokines in response to various inflammatory stimuli (Ge et al., 2024).

m^6^A methylation governs sepsis progression by modulating pro-inflammatory cytokine expression and regulating immune-cell activation and cytokine secretion (Shen et al., 2023). The interdependence between m^6^A methylation and inflammatory response is well-established (Song et al., 2023; Luo et al., 2021a). The NOD-like receptor family pyrin domain containing 3(NLRP3) inflammasome has been mechanistically linked to septic pathology (Zhang W. et al., 2023). Using Lipopolysaccharide (LPS)-induced septic shock models, Luo et al. showed that FTO inhibition suppresses NLRP3 inflammasome activation through the forkhead box protein O1(FoxO1)/NF-κB signaling pathway in macrophages (Luo et al., 2021b). Correspondingly, Wang et al. revealed that modulating FTO-mediated m^6^A methylation regulates pyroptosis in sepsis (Wang B. et al., 2022)—a key mechanism driving uncontrolled inflammation. Thus, m^6^A methylation not only contributes significantly to septic pathogenesis but also represents a promising immunomodulatory target (Figure 2).

Furthermore, Hotchkiss’ proposition of sepsis as an immunological disorder is supported by autopsy evidence demonstrating immune-cell depletion via apoptosis in deceased patients (Hotchkiss et al., 2013b). Importantly, m^6^A dysregulation impairs macrophage phagocytic function and disrupts neutrophil chemotaxis, representing critical factors in septic pathology progression (Qian and Cao, 2022). Experimental studies in severe sepsis models indicate that YTHDF1 knockdown alleviates macrophage paralysis and endothelial damage. Mechanistically, YTHDF1 functions as an m^6^A reader that recognizes m^6^A modifications on JAK2/STAT3 mRNA and promotes its translation, thereby enhancing JAK-STAT signaling activity. When YTHDF1 is knocked down, its translational enhancement of JAK2/STAT3 mRNA is weakened, resulting in reduced JAK2/STAT3 protein expression (including phosphorylated forms) (Xing et al., 2021). Additionally, m^6^A methylation mediates negative regulation of serine protease inhibitor 2A (*Spi2a*) in macrophages, consequently inhibiting the release of pivotal pro-inflammatory cytokines such as tumor necrosis factor-α(TNF-α) and interleukin-6(IL-6), which are central to septic inflammatory cascades (Wang et al., 2023).

## 4 Role of m^6^A methylation in sepsis-induced organ dysfunction

**FIGURE 3:**
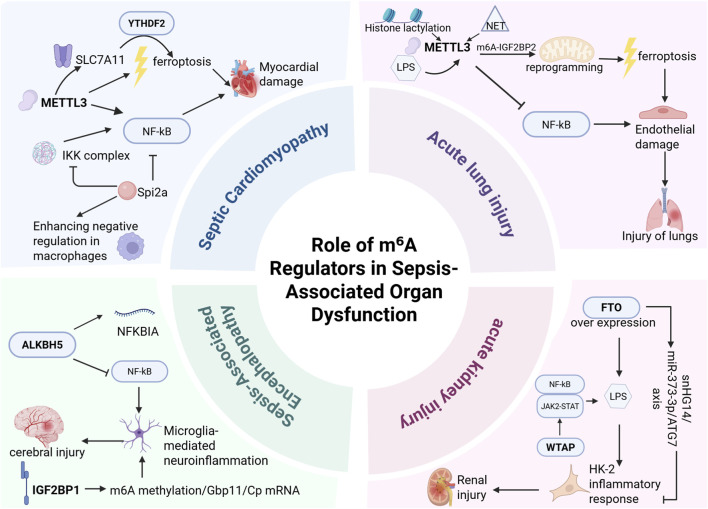
m^6^A modification plays a critical role in sepsis-associated organ injury, primarily involving sepsis-induced cardiomyopathy (SCM), acute lung injury (ALI), sepsis-associated encephalopathy (SAE), and acute kidney injury (AKI). In SCM, METTL3 exacerbates multi-organ dysfunction by promoting cardiomyocyte ferroptosis and NF-κB activation. In ALI, METTL3 augments m^6^A-IGF2BP2-dependent mitochondrial metabolic reprogramming to intensify ferroptosis while simultaneously regulating endothelial function through Trim59-mediated NF-κB inactivation, demonstrating high diagnostic and therapeutic value. In SAE, ALKBH5 inhibits NF-κB pathway activation to mitigate microglia-mediated neuroinflammation; IGF2BP1 may regulate microglial inflammatory responses by enhancing m^6^A methylation and stabilizing Gbp11/Cp mRNAs, emerging as a potential therapeutic target for microglial hyperactivation. In AKI, FTO ameliorates renal injury by suppressing autophagy, reducing RNA stability, and downregulating SNHG14 expression, whereas WTAP promotes LPS-induced inflammation and renal damage in HK-2 cells via NF-κB and JAK2/STAT3 pathway regulation. These findings highlight the significance of m^6^A regulators as potential therapeutic targets for combating sepsis-induced organ damage.

MODS represents a severe dysregulated systemic inflammatory state triggered by sepsis, and is characterized by progressive functional deterioration or failure of two or more vital organ systems (Shi et al., 2019) (e.g., heart, lungs, kidneys). As the terminal stage of sepsis, MODS carries a mortality rate of 28%–56% upon diagnosis (Zou et al., 2022). Emerging evidence indicates that m^6^A methylation modulates sepsis progression through multiple pathways, playing a pivotal role in MODS development (Shen et al., 2023; Zhang S. et al., 2022) (Figure 3). Consequently, elucidating m^6^A’s functions in sepsis-induced organ dysfunction is crucial for optimizing clinical management and developing novel therapeutics.

### 4.1 m^6^A methylation and myocardial injury

Septic cardiomyopathy (SCM), a non-ischemic cardiac dysfunction in sepsis, features impaired left/right ventricular systolic or diastolic function, accompanied by cardiomyocyte damage and inflammation-driven pathophysiological alterations (Beesley et al., 2018). Inflammatory cytokines (e.g., IL-6, TNF-α) directly induce cardiomyocyte dysfunction through oxidative stress, calcium mishandling, and mitochondrial damage, leading to hemodynamic instability—manifested as tachycardia, reduced cardiac output, and impaired contractility. These changes exacerbate myocardial ischemia-hypoxia, creating a vicious cycle of injury (Bi et al., 2023).

Recent studies reveal that m^6^A methylation regulates septic myocardial injury by modulating inflammation and apoptosis. Wang et al. demonstrated METTL3’s protective role in murine sepsis models, where METTL3 inhibition exacerbated multi-organ damage (Wang et al., 2024). Shen et al. further validated METTL3’s interaction with solute carrier family seven member 11 (*Slc7a11*) via RIP-qPCR and MeRIP-qPCR, showing elevated METTL3 expression and methylation levels in LPS-treated rat cardiomyocytes. METTL3 promotes *Slc7a11* mRNA degradation through m^6^A-dependent mechanisms, intensifying sepsis-induced cardiomyocyte ferroptosis—an iron-dependent, lipid per oxidation-mediated cell death strongly implicated in sepsis pathogenesis. This establishes the METTL3/YTHDF2/*Slc7a11* axis as central to septic myocardial injury (Shen et al., 2023).

Supporting this, Tang’s team found that METTL3 silencing suppressed ferroptosis in septic rat cardiomyocytes via *Slc7a11* m^6^A methylation (Tang et al., 2024). Complementarily, Zhang reported that in a mouse model of sepsis, YTHDF1 can inhibit pyroptosis of cells and alleviate the damage caused by sepsis by promoting the ubiquitination of NLRP3 and upregulating the WW domain-containing E3 ubiquitin ligase 1 (*Wwp1*) (Zhang S. et al., 2022). Wang et al. Identified *Spi2a* as a novel negative feedback regulator that suppresses cytokine production and myocardial injury in macrophages post LPS challenge by inhibiting inhibitor of kappa B kinase (IKK) complex formation and NF-κB activation. Critically, they proved *Spi2a*’s m^6^A methylation sustains macrophage feedback control. Through comprehensive experiments (on cellular, animal, molecular, and clinical specimens), According to Wang et al., the METTL3/METTL14 complex synergistically enhances *Spi2a* mRNA stability and translation through m^6^A modification. METTL3 provides the catalytic activity for methylation, while METTL14 stabilizes the complex and enhances substrate recognition. The m^6^A-modified *Spi2a* mRNA is then recognized by YTHDF1, which promotes its translational efficiency. This mechanism leads to increased SPI2A expression, subsequently suppressing IKKβ/NF-κB-mediated inflammation (Wang et al., 2023). This indicates that m^6^A orchestrates SCM pathology at multicellular levels through distinct targets (e.g.,.*Spi2a* in macrophages), uncovering novel therapeutic avenues that target m^6^A modifiers (e.g., METTL3, METTL14, or SPI2A). Additionally, Shen et al. implicated METTL3 in septic rat myocardial injury via the METTL3/IGF2BP1/m^6^A/HDAC4 axis (Shen et al., 2022). Collectively, METTL3 and YTHDF1 emerge as promising diagnostic and therapeutic targets.

### 4.2 m^6^A methylation and lung injury

The lungs are highly susceptible to sepsis, and acute respiratory distress syndrome (ARDS) and acute lung injury (ALI) serve as critical prognostic indicators (Wu et al., 2024). ARDS affects 10.4% of ICU patients and 23.4% of mechanically ventilated cases, with overall mortality at 40% (mild: 34.9%; moderate: 40.3%; severe: 46.1%) (Wick et al., 2024). Pathologically, ARDS/ALI features endothelial damage and dysregulated innate immunity. Polymorphonuclear neutrophils (PMNs) and platelets play pivotal roles: recruited PMNs eliminate pathogens via degranulation, phagocytosis, and neutrophil extracellular trap (NET) formation. NETs—extracellular networks of DNA, histones, myeloperoxidase (MPO), cathepsin G, and antimicrobial proteins—neutralize pathogens but paradoxically propagate inflammation and tissue damage when overproduced (Silva et al., 2021; Ma et al., 2017). Studies indicate that enhanced formation of NETs in sepsis-associated ALI/ARDS activates METTL3-mediated m6A modification in alveolar epithelial cells, which regulates the stability of HIF-1α, thereby inducing mitochondrial metabolic reprogramming and ferroptosis, ultimately leading to lung injury.

Mounting evidence implicates METTL3 in sepsis-induced ALI. Ferroptosis (Zhang H. et al., 2023; Lai et al., 2025)—an iron-dependent cell death driven by uncontrolled lipid peroxidation—emerges as a key mechanism (Jiang et al., 2021b). Zhang et al. demonstrated elevated NETs in cecal ligation and puncture (CLP)-induced ALI mice, and showed that NET inhibitors reversed ferroptosis. Integrated RNA-seq and MeRIP-seq revealed that NET-induced METTL3 upregulation exacerbates ferroptosis in alveolar epithelium via m^6^A-Insulin Like Growth Factor 2 MRNA Binding Protein 2(IGF2BP2)-dependent mitochondrial metabolic reprogramming, thereby offering therapeutic targets to mitigate lung injury and systemic inflammation (Zhang H. et al., 2023). Experiments in Zhang et al.'s laboratory further corroborated this phenomenon (Zhang H. et al., 2022). Chen et al. initially detected reduced global m^6^A levels in septic patients through colorimetric ELISA assays. Subsequent Western blotting analysis revealed significantly diminished METTL3 expression in the lung tissues of these patients compared to healthy controls, suggesting a potential association between METTL3 dysregulation and sepsis pathogenesis. The team conducted *in vivo* experiments using METTL3-knockdown murine models *versus* wild-type counterparts, and demonstrated that METTL3 deficiency exacerbated endothelial barrier disruption, amplified sepsis-induced inflammatory responses, and consequently aggravated pulmonary injury. For *in vitro* validation, they employed transfection techniques to inhibit METTL3 in LPS-stimulated HULEC-5a cells across multiple time points, and observed impaired endothelial permeability and intensified barrier dysfunction. Furthermore, METTL3 was found to modulate endothelial function in sepsis-induced acute lung injury by inactivating NF-κB through Tripartite Motif Containing 59 (Trim59)-mediated mechanisms (Chen et al., 2022).

Wu et al. validated that histone lactylation induces METTL3-mediated m^6^A modification to promote ferroptosis (Wu et al., 2024), identifying METTL3 targeting as a viable strategy against septic lung injury. Notably, this regulatory axis exemplifies a broader and highly significant “epigenetic hierarchical network” in sepsis pathogenesis, where upstream histone post-translational modifications (PTMs)orchestrate downstream RNA epigenetic modifications (like m^6^A) to coordinately amplify the inflammatory response. For instance, metabolic reprogramming during sepsis leads to lactate accumulation, which drives histone lactylation to upregulate METTL3 expression; the increased METTL3 then deposits m^6^A marks on pro-inflammatory transcripts, enhancing their stability and translation efficiency and further fueling inflammation and lactate production. This creates a positive feed-forward loop that potently exacerbates the cytokine storm and organ damage. Recognizing such multi-layered epigenetic crosstalk not only deepens our mechanistic understanding of septic inflammation but also opens new avenues for therapeutic intervention. Similarly,Lai et al. established an LPS-stimulated human pulmonary microvascular endothelial-cell (HPMEC) model showing that METTL14/IGF2BP2-mediated m^6^A modification of *STEAP1* aggravates ALI (62). Complementarily, Xian et al. reported macrophage NLRP3 inflammasome hyperactivation during ALI/ARDS progression (Xian et al., 2021; Cao et al., 2024). Building on this, Cao’s team identified *Nlrp3* as a METTL14 target. They demonstrated that knockdown of IGF2BP2 reduces LPS-induced ALI by downregulating *Nlrp3* expression, achieved through a decrease in *Nlrp3* transcript stability and inhibition of the *Nlrp3* inflammasome activation, thereby highlighting METTL14’s therapeutic potential. Collectively, these findings have transformative potential for advancing diagnostic biomarkers, therapeutic strategies, and prognostic evaluation in sepsis management (Cao et al., 2024).

### 4.3 m^6^A methylation and brain injury

Sepsis-associated encephalopathy (SAE), a frequent neurological complication of sepsis, manifests as brain dysfunction and neuronal damage during systemic inflammation, characterized by delirium, disturbances of consciousness, and cognitive impairment (Bircak-Kuchtova et al., 2023). Emerging evidence implicates m^6^A methyltransferases in SAE pathogenesis (Ye et al., 2025; Wang H. et al., 2022; Ding et al., 2022; Li et al., 2021; Mittal and Coopersmith, 2014). Wang and colleagues detected serum markers using the enzyme-linked immunosorbent assay method. They found that compared with non-SAE patients, the expression of METTL3 was significantly increased in SAE patients, while the expression of FTO was significantly decreased. (Wang H. et al., 2022).

While microglia in the resting state primarily maintain normal central nervous system function, their excessive activation may contribute to the onset and pathology of SAE. Ye et al. demonstrated through both mechanistic and clinical validation that in a murine model of sepsis, ALKBH5-mediated m^6^A demethylation stabilizes NF-κB inhibitor alpha (*Nfkbia*) mRNA, thereby elevating NFKBIA protein levels, suppressing p65 phosphorylation and nuclear translocation, inhibiting the NF-κB signaling pathway, and ultimately alleviating microglia-mediated neuroinflammation; furthermore, in human sepsis patient samples, ALKBH5 expression was found to correlate with disease severity. (Ye et al., 2025). Complementarily, Ding et al. identified IGF2BP1 as a regulator of microglial inflammation in mouse primary microglia through m^6^A-dependent stabilization of Guanylate Binding Protein 1 (Gbp11) and Cp mRNAs. They proposed IGF2BP1 inhibition as a strategy to mitigate microglial hyperactivation (Ding et al., 2022). Li et al., using primary microglia isolated from newborn (<24 h) Sprague-Dawley (SD) rat brains, further mapped differential m^6^A modifications in M0-like (resting), M1-like (pro-inflammatory), and M2-like (anti-inflammatory) microglial subtypes, establishing m^6^A as a key modulator during microglial immune responses. (Li et al., 2021). These collective findings underscore m^6^A’s role in regulating microglial inflammatory states, and clarify its direct impact on SAE progression and outcomes.

Intriguingly, Wang et al. integrated LC-MS/MS metabolomics and 16S rDNA sequencing to identify gut microbiota dysbiosis in SAE, and detected the expression of serum markers and IL-6 by enzyme-linked immunosorbent assay (ELISA). Comparative analysis of gut microbiota between SAE and non-SAE cohorts revealed a positive correlation between *Acinetobacter* abundance and METTL3 upregulation. This indicated that targeted METTL3 modulation could restore microbial homeostasis, thereby ameliorating or even therapeutically resolving SAE pathology (Wang H. et al., 2022).

### 4.4 m^6^A methylation and kidney injury

Acute kidney injury (AKI) frequently complicates sepsis through pathological mechanisms including microcirculatory dysfunction, dysregulated immune responses, coagulation activation, and renal tubular epithelial damage (Adedoyin et al., 2018). Clinically manifested as abrupt loss of kidney function with oliguria and elevated serum creatinine, sepsis associated-acute kidney injury (SA-AKI) affects >40% of septic patients and represents a major independent risk factor for ICU mortality (Martin et al., 2016; Naime et al., 2018; Alanazi et al., 2024). Current therapeutic strategies—including antimicrobial therapy, fluid resuscitation, vasoactive agents, and renal replacement therapy—demonstrate limited efficacy. Emerging research implicates m^6^A methylation in regulating ferroptosis during AKI pathogenesis, with METTL14 appearing to be a pivotal regulator of ferroptosis in renal disease progression (Adedoyin et al., 2018; Yang et al., 2024).

Small nucleolar RNA host gene 14(*SNHG14*) exacerbates renal injury by activating microglia and modulating the *miR-373-3p/ATG7* axis in LPS-stimulated HK-2 cells (Yang et al., 2024; Yang N. et al., 2021; Tan et al., 2020). Yang et al. demonstrated that FTO confers nephroprotection in sepsis patients with acute kidney injury (AKI) by suppressing autophagy through RNA destabilization and reduced *SNHG14* expression, thus mitigating LPS-induced renal damage. (Yang et al., 2024). Huang et al., using an AKI mouse model established by cecal ligation and puncture (CLP) and an AKI cell model established by treating HK-2 cells with LPS, reported that *Wtap* knockdown promotes inflammation, ferroptosis, and cellular injury in LPS-treated HK-2 cells by upregulating lamin B1 (*Lmnb1*) expression while activating NF-κB and JAK2/STAT3 signaling pathways. (Huang et al., 2024). Complementary to these findings, Xu et al., using TCMK-1 cells to establish *in vitro* AKI models and LPS-treated mice for *in vivo* AKI models, observed rapid m^6^A elevation in LPS-challenged murine renal epithelial (TCMK-1) cells. Notably, METTL14 knockdown counteracts LPS-aggravated ferroptosis in these *in vivo* murine models. (Xu L. et al., 2025).

Collectively, inhibition of METTL14 alleviates both renal injury and ferroptosis in LPS-induced AKI, establishing m^6^A methylation as a pivotal therapeutic target for future AKI interventions.

### 4.5 m^6^A methylation and other organ injuries

The liver critically regulates systemic immune responses during sepsis by means of bacterial clearance, cytokine production, and metabolic adaptations to inflammation (Sun et al., 2020). However, sepsis-induced ischemic hepatic injury, shock-related damage, and secondary sclerosing cholangitis collectively establish the liver as a primary target of sepsis-mediated secondary injury (Strnad et al., 2017). As an independent predictor of ICU outcomes, identifying therapeutic targets for septic liver injury is imperative (Wang et al., 2025). Sun et al. demonstrated that in septic mice,IGF2BP3 interacts with GLI family zinc finger 2 (GLI2) mRNA to stabilize m^6^A-modified transcripts. Upregulated *Gli2* transcriptionally promotes synoviolin 1 (*Syvn1*) expression, which subsequently enhances degradation of peroxisome proliferator-activated receptor alpha (PPARα). This cascade ultimately exacerbates septic liver injury both *in vitro* and *in vivo* by suppressing PPARα-mediated autophagy, establishing the IGF2BP3/GLI2/*Syvn1*/PPARα axis as a potential therapeutic target (Sun et al., 2024).

In summary, current research demonstrates that m^6^A RNA methylation—orchestrated through the dynamic interplay of Writers, Erasers, and Readers—precisely regulates key signaling pathways involved in inflammation, apoptosis, and autophagy. This epigenetic mechanism serves as a central driver of inflammatory amplification, tissue-barrier disruption, and cellular dysfunction during sepsis-induced secondary organ injury (Table 2). These findings establish critical targets and pathways for therapeutic intervention while opening novel directions for clinical translation.

**TABLE 2:** Mechanism of m6A RNA Modification in the Context of Sepsis-Induced Organ Damage.

Disease	Factor	Type	Intervention method	Expression alteration	Relevant targets	Function	References
SCM	METTL3	Writer	Inhibition	Upregulation	TNF-α	Modulating TNF-α release to aggravate inflammation	(Wang et al., 2024)
	METTL3	Writer	Knockdown	Downregulation	SLC7A11 mRNA	Alleviate LPS-induced ferroptosis	(Shen et al., 2023)
	METTL3	Writer	Knockdown	Downregulation	HDAC4 mRNA	Alleviate LPS-induced ferroptosis	(Shen et al., 2022)
	METTL3	Writer	Knockdown	Downregulation	SLC7A11	Suppress ferroptosis	(Tang et al., 2024)
	METTL14/METTL3complex	Writer	KAT2B-mediated acetylation of METTL14	Downregulation	IKKβ/NF-κB	Enhance the expression of Spi2a and inhibit the NF-κB pathway	(Wang et al., 2023)
	YTHDF1	Reader	Overexpression	Downregulation	WWP1	Enhance NLRP3 ubiquitination and inhibit pyroptosis	(Zhang et al., 2022a)
ARDS/ALI	METTL3	Writer	Overexpression	Downregulation	Trim59	Trim59-associated NF-κB inactivation	(Chen et al., 2022)
	METTL3	Writer	Knockdown	Downregulation		The PAD4/NETs/METTL3 axis	Zhang et al. (2023b), Zhang et al. (2022b)
	METTL3	Writer	Short-term lactate stimulation	Upregulation		The GPR81/H3K18la/METTL3/ACSL4 axis	(Wu et al., 2024)
	METTL14/IGF2BP2	Writer; Reader		Upregulation	STEAP1 mRNA	Stabilized STEAP1 mRNA expression	(Lai et al., 2025)
	METTL14	Writer	Knockdown	Downregulation	NLRP3	Inhibit the activation of NLRP3 inflammasome	(Cao et al., 2024)
SAE	ALKBH5	Eraser	Overexpression	Downregulation	NFKBIA	Inhibition of the NF-κB inflammatory signal pathway	(Ye et al., 2025)
	IGF2BP1	Reader		Unspecified	Gbp11; Cp mRNA	Enhance the m6A methylation and stability of Gbp11 and Cp mRNAs	(Ding et al., 2022)
AKI	FTO	Eraser	Overexpression	Downregulation	SNHG14	Decrease the RNA stability and expression of SNHG14	(Yang et al., 2024)
	WTAP	Writer	Knockdown	Downregulation	LMNB1	NF-κB and JAK2/STAT3 pathways	(Huang et al., 2024)
	METTL14	Writer	Knockdown	Downregulation	LPCAT3	LPCAT3 overexpression antagonizes ferroptosis	(Xu et al., 2025b)
Sepsis liver injury	IGF2BP3	Reader	Knockdown	Downregulation	GLI2 mRNA	Inhibition of the GLI2/SYVN1/PPARα axis	(Sun et al., 2024)

## 5 Conclusion and perspectives

Sepsis-induced multi-organ injury involves complex pathogenic networks. This review has examined mechanisms underlying sepsis-mediated organ damage and delineated the regulatory roles of m^6^A methylation: Writer, Eraser, and Reader proteins participate dynamically in critical biological processes by post-transcriptionally modulating cellular gene expression, thereby propagating secondary organ injury. These modifications influence RNA fate through splicing, transport, translation, stabilization, and degradation, profoundly impacting sepsis progression.

mRNA methylation and its regulators exhibit broad biological functions. Notably, certain regulators such as METTL3/YTHDF2 exacerbate cellular damage by amplifying inflammatory pathways, while FTO/ALKBH5 confer protective effects by destabilizing pro-inflammatory cytokine mRNAs. Interactions with non-coding RNAs further form regulatory networks influencing sepsis progression. These discoveries provide novel therapeutic insights into organ-specific damage in sepsis. The therapeutic potential of targeting the m^6^A machinery could be realized through several strategic approaches: 1) Developing small-molecule inhibitors against “Writer” complexes (e.g., METTL3/METTL14) or “Erasers” (e.g., FTO, ALKBH5) to globally reduce or selectively reshape the m^6^A epitranscriptome; 2) Designing compounds that disrupt the interaction between specific “Reader” proteins (e.g., YTHDF2) and their pro-inflammatory target mRNAs, offering a more precise intervention; 3) Exploiting upstream regulatory cues, such as modulating the lactate-induced histone lactylation that drives METTL3 expression, to indirectly influence m^6^A deposition; 4) Exploring combination therapies where m^6^A-targeting agents are used alongside conventional antibiotics or specific pathway inhibitors to achieve synergistic effects and overcome immunosuppression.

We recognize that targeting ubiquitously expressed enzymes like METTL3 presents a specificity challenge, which is reflected in their context-dependent roles across different organs. For instance, METTL3 exacerbates injury in cardiomyocytes and alveolar epithelial cells by promoting ferroptosis, whereas in pulmonary endothelial cells and the gut, it exhibits protective effects by maintaining barrier integrity and modulating inflammatory responses. This apparent contradiction is not a paradox but can be explained by an emerging paradigm: m^6^A regulates sepsis through several evolutionarily conserved, cross-organ pathways—primarily by amplifying inflammatory signaling, programmed cell death, and metabolic reprogramming, which collectively drive the pathological process. The key insight is that the same pathway (e.g., NF-κB or ferroptosis) may produce opposing outcomes in different tissues due to cell-type-specific molecular targets. For example, METTL3-mediated m^6^A modification promotes NF-κB activation in macrophages (Wang et al., 2023), yet suppresses it in pulmonary endothelial cells via Trim59 (65). Similarly, while ferroptosis is universally pathogenic, its triggering mechanisms vary significantly. This paradigm reveals that the core pathways are shared, but the cellular context determines the final, organ-specific effects.

Our understanding of METTL3 and METTL14 in sepsis is currently confined to their m^6^A-dependent functions, this emerging paradigm from other fields highlights a critical, non-canonical dimension of their functionality. The findings by Dou et al. and Liu et al. provide a foundational rationale for hypothesizing that METTL3 may act as a transcriptional co-activator on inflammatory gene promoters, (Liu et al., 2021), while METTL14 may engage in direct chromatin regulation, as exemplified by its interaction with H3K27me3 and recruitment of KDM6B (Dou et al., 2023), provides a mechanistic precedent for METTL14 acting beyond the Methyltransferase Complex (MTC).

As a pivotal RNA modification, m^6^A methylation has garnered substantial research interest in sepsis-related organ injury in recent years. Despite extensive investigations into its roles in sepsis, the precise regulatory mechanisms remain incompletely elucidated, which presents ongoing challenges. Key knowledge gaps include: undefined interactions among m^6^A regulatory factors during sepsis; potential organ-specific regulatory factors within m^6^A networks that may explain injury heterogeneity (with such factors potentially serving as novel biomarkers for sepsis severity, organ-injury risk, and treatment prognosis); Translating these findings into clinical applications faces significant hurdles. Currently, no clinical trials are specifically investigating m^6^A-targeted therapies for sepsis or infectious diseases, underscoring the nascent stage of this field. The path to clinical translation is fraught with challenges, primarily due to the context-dependent nature of m^6^A function, which varies by cell type, pathological phase, and target gene, raising concerns about therapeutic specificity and potential off-target effects. Furthermore, achieving organ- or cell-selective drug delivery remains a major pharmacological bottleneck. Lastly, the essential physiological roles of m^6^A regulators necessitate a careful assessment of the safety profile and a narrow therapeutic window in critically ill septic patients. Crucially, most current conclusions are derived from murine and *in vitro* models, which means that clinical studies in sepsis patients are needed to validate the relationships between m^6^A dysregulation and secondary organ damage.

In summary, targeting m^6^A regulators holds great potential for sepsis diagnosis, treatment, and prognosis, yet comprehensive research remains essential to fully harness their therapeutic capabilities.
